# Diabetic uterus environment may play a key role in alterations of DNA methylation of several imprinted genes at mid-gestation in mice

**DOI:** 10.1186/1477-7827-11-119

**Published:** 2013-12-30

**Authors:** Zhao-Jia Ge, Qiu-Xia Liang, Shi-Ming Luo, Yan-Chang Wei, Zhi-Ming Han, Heide Schatten, Qing-Yuan Sun, Cui-Lian Zhang

**Affiliations:** 1 Reproductive Medicine Center, Henan Provincial People’s Hospital, Zhengzhou 450003, Henan Province, P. R. China; 2 Reproductive Medicine Center, People’s Hospital of Zhengzhou University, Zhengzhou 450003, Henan Province, P. R. China; 3 State Key Laboratory of Reproductive Biology, Institute of Zoology, Chinese Academy of Sciences, Beijing 100101, P. R. China; 4 Department of Veterinary Pathobiology, University of Missouri, Columbia, MO 65211, USA

**Keywords:** Maternal diabetes milieu, DNA imprinting, Placenta

## Abstract

**Background:**

Maternal diabetes mellitus not only has severe deleterious effects on fetal development, but also it affects transmission to the next generation. However, the underlying mechanisms for these effects are still not clear.

**Methods:**

We investigated the methylation patterns and expressions of the imprinted genes *Peg3*, *Snrpn*, and *H19* in mid-gestational placental tissues and on the whole fetus utilizing the streptozotocin (STZ)-induced hyperglycemic mouse model for quantitative analysis of methylation by PCR and quantitative real-time PCR. The protein expression of *Peg3* was evaluated by Western blot.

**Results:**

We found that the expression of *H19* was significantly increased, while the expression of *Peg3* was significantly decreased in dpc10.5 placentas of diabetic mice. We further found that the methylation level of *Peg3* was increased and that of *H19* was reduced in dpc10.5 placentas of diabetic mice. When pronuclear embryos of normal females were transferred to normal/diabetic (NN/ND) pseudopregnant females, the methylation and expression of *Peg3* in placentas was also clearly altered in the ND group compared to the NN group. However, when the pronuclear embryos of diabetic female were transferred to normal pesudopregnant female mice (DN), the methylation and expression of *Peg3* and *H19* in dpc10.5 placentas was similar between the two groups.

**Conclusions:**

We suggest that the effects of maternal diabetes on imprinted genes may primarily be caused by the adverse uterus environment.

## Background

Infants of mothers with pre-existing types 1, 2 or gestational diabetes have significantly higher rates of perinatal mortality and major congenital anomaly [1,2]. Diabetic embryopathy can affect many organ systems including the heart and the neural tube [3-5]. The effects of maternal diabetes mellitus on fetal development have been studied in various animal models. In drug-induced animal models, embryo development was disturbed by maternal hyperglycaemia [6,7]. In non-obese diabetic (NOD) mouse models of spontaneous type 1 diabetes mellitus, only 20% of recovered NOD embryos reached the blastocyst stage at 96 hours of superovulation compared with 90% of embryos recovered from nondiabetic animals [6].

Several reports have shown that teratogenesis may be a direct result of hyperglycemia [8,9]. Previous studies found that expressions of genes related to metabolism, development and signal transduction were altered in embryos by the diabetic environment [10,11]. Moley *et al*. showed that the mRNA and protein expression of the glucose transporters GLUT-1, GLUT-2, and GLUT-3 were decreased in embryos from streptozotocin (STZ)-induced hyperglycaemic mice [12]. These results partly elucidate how maternal diabetes mellitus causes abnormal embryo development. But it is still not clear how the adverse effects are inherited to the next generations.

Wellen *et al.* found that excessive glucose affected histone acetylation via the citrate lyase pathway [13]. This finding suggests the possibility that the epigenome of the embryo may contribute significantly to abnormal fetal development in diabetic females. DNA methylation, one of the epigenetic modifications on DNA, can regulate relative gene expression, X-chromosome inactivation, as well as genomic imprinting [14]. Genomic imprinting includes the formation of DNA methylation at specific loci in a parent-of-origin-specific manner [15]. If DNA methylation on imprinted genes is not acquired/maintained properly, embryonic development and the offspring’s health would be affected [16,17].

The DNA methylation pattern of imprinted genes is susceptible to being affected by the environment [18]. Several reports have shown that pre-implantation culture and manipulation can cause an abnormal methylation status of Differentially Methylated Regions (DMRs) at imprinted loci and these changes may induce abnormal fetal development [19-21]. If maternal nutrients are altered the DNA methylation patterns may also be changed and this will induce abnormalities during fetal development. During gestation, if female rats are fed with choline-deficient diets, the DNA methylation of G9a and Suv39h1 is mis-regulated [22]. These data indicate that the adverse maternal environment exerts adverse effects on DNA methylation during genomic imprinting establishment and maintenance.

We hypothesized that impaired DNA methylation at imprinted loci may play a key role in causing abnormal embryo development in maternal diabetes mellitus. In our lab, we have found that the DNA methylation patterns in DMRs of imprinted genes *Peg3, Snrpn* and *H19* in oocytes was not altered by maternal diabetes at 15 days of injection of STZ [23], but the embryonic development was affected. This indicated that the uterus environment may have deleterious effects on embryonic development. We examined the methylation patterns of DMRs of *Peg3, Snrpn,* and *H19* in day post-coitum (dpc)10.5 placenta and fetus in the STZ-induced mouse model. We found that the expression and methylation levels of the imprinted genes were altered by maternal diabetes mellitus in placentas at 10.5dpc of gestation. Previous studies have shown that if the pre-gestational type 1 diabetes mellitus was cured at pre-pregnancy, the risks of adverse pregnancy outcomes was reduced in women [24]. In animal models, if diabetic females were treated with insulin, embryonic development was not significantly different from that in non-diabetic females [6]. Therefore, we also investigated whether the adverse effects caused by maternal diabetes on imprinted genes in placentas could be corrected by embryo transfer.

## Methods

### Ethics statement

All procedures described were reviewed and approved by the ethical committee of the Institute of Zoology, Chinese Academy of Sciences.

All mice were provided by the Beijing Vital River Experimental Animals Centre and fed in a temperature controlled room with a light cycle of 12 L: 12D (light:dark).

### Generation of the diabetic mouse model

Female CD-1® (strain code; 022) mice, aged 6–7 weeks, received a single intraperitoneal injection of streptozotocin (STZ) at a dose of 230 mg/kg [25]. Four days later, blood glucose levels were checked using a glucometer, Blood Testing Equipment, Accu-CHEK Active (Roche Diagnostic, Germany). If glucose levels were higher than 17.0 mmol/l, the mice were selected and used as the diabetic model (diabetic mice n = 53). Mice of similar age injected with buffer were selected as control (non-diabetic mice n = 49).

### Collecting placentas and fetuses at mid-gestation

Diabetic/nondiabetic mice were mated naturally with normal male mice within 15 days of STZ/buffer injection and were determined to be pregnant when the vaginal plug was examined at 0.5d. At 10.5d of gestation, placentas (trophoblast population) and whole fetuses were collected. Samples were frozen immediately in liquid nitrogen and stored at -80°C until used.

### DNA purification and quantitative analysis of methylation by PCR (qAMP)

DNA was purified from one half of the bisected fetus or placentas using DNA Tissue Kit (Tiangen, China) following the manufacturer’s directions. To investigate the methylation conditions, qAMP was conducted as described by Oakes *et al.* and Lopes *et al.*[26,27]. Briefly, fetal or placental DNA (~1.5 μg/enzyme) was digested with methylation-sensitive (*Hha*I*,* New England Biolabs, Beijing, China; *Hpa*II, New England Biolabs, Beijing, China) and methylation-dependent (*McrBc*, New England Biolabs, Beijing, China) restriction enzymes, respectively. The resulting product was used as template for real-time PCR reaction using the SYBR green kit (Kangwei Inc., China). The qAMP was performed employing a Rotor-Gene Q Real-Time PCR instrument (Qiagen). The percentage of methylation for cytosine-phosphate-guanine (CpG) sites was based on ⊿Ct (cycle threshold values between digested and undigested sham aliquots). For *HhaI*, the percent methylation is 100 (e^-0.7(∆Ct)^); for *McrBC*, the percent methylation is 100 (1- e^-0.7(∆Ct)^). The primers described in Table 1 were designed to span ~ 180 bps of the DMRs of the maternally methylated sequences: *Peg3* and *Snrpn*, as well as the paternally methylated *H19*.

**Table 1:** Oligonucleotides and annealing temperature utilized for qAMP and qRT-PCR of imprinted genes

		**Primer sequence**	
		**Forward**	**Reverse**
**qAMP**	***H19***	5′-AGCCGTTGTGAGTGGAAAGA-3′	5′-CATAGCGGCTTCGGACATT-3′
	***Snrpn***	5′-CTCCTCAGAACCAAGCGTCT-3′	5′-ATTCCGGTCAGAGGGACAGA-3′
	***Peg3***	5′-GGTGTCCCGCAGCCCTTG-3′	5′-CGGAGCACAGCACTCTACGC-3′
	chr9:106724005-106724149	5′-GATCTATTCCTTCCTTTACTTT-3′	5′-TCCTGGGAAATGAAGTTT-3′
***Peg3***	**qRT-PCR**	5′-TTGGACTGGACAGAGATGATGACA-3′	5′-ATTCTGGTATGACTCGGCATCCT-3′
***Snrpn***		5′-AGGCCCATCCCAGCAGGTCAT-3′	5′-GCGGGTACTGGGTTGGGGCTC-3′
***H19***		5′- CTTGTCGTAGAAGCCGTCTGTTC-3′	5′- GTAGCACCATTTCTTTCATCTTGAGG-3′
***Ppia***		5′- CGCGTCTCCTTCGAGCTGTTTG-3′	5′- TGTAAAGTCACCACCCTGGCACAT-3′

### RNA isolation and mRNA expression assays

RNA was extracted from the other half of the bisected fetus and placentas using RNA Tissue kit (Tiangen, China) according to the manufacturer’s instructions. cDNA first-strand synthesis was performed using Superscript II (Invitrogen). The first-strand cDNA was used as template and the expressions of *Peg3, Snrpn* and *H19* were evaluated by quantitative real-time PCR (qRT-PCR) using Rotor-gen Q (Qiagen, Germany). Triple reactions were analyzed for each sample and the threshold cycle value was normalized to the housekeeping gene of peptidylprolyl isomerase A (*Ppia*). The primers are shown in Table 1. The expression levels were calculated using the 2^-△△Ct^ method.

### Western blot and immunohistochemistry analysis

Placental proteins (n = 6 from 4 litters) were extracted from cell lysis and quantified for western blot analysis as previously described [28]. Briefly, proteins were heated for 5 min at 100°C. Then proteins were separated by SDS-PAGE and electrically transferred to polyvinylidene fluoride membranes. After that, the membranes were blocked in TBST containing 5% skimmed milk for 2 h, followed by incubation overnight at 4°C with rabbit anti-peg3 (1:500, Bioss, China, code: bs-1870R). After washed with TBST, membranes were incubated with goat anti-rabbit IgG (1:1000) for 1 h at 37°C. Beta-actin was used as loading control.

For immunohistochemistry analysis, fresh placentas (n = 6 from 4 litters) were fixed in 4% paraformaldehyde overnight at 4°C and washed in 50% ethanol for 1 hour at room temperature. After that, samples were stored in 70% ethanol at 4°C until used. The fixed samples were dehydrated in a graded ethanol series, cleared in xylene, and embedded in paraffin wax. The embedded placentas were sectioned at 8 μm and incubated with rabbit anti-Peg3 (1:500, Bioss, China, code: bs-1870R) overnight at 4°C. The samples were then incubated with the biotin-labeled secondary antibody for 30 minutes. Staining was carried out using the Vectastain ABC kit and DAB peroxidase substrate kit (Vector Laboratories, Burlingame, CA).

### Embryo transfer

To obtain pronuclear stage embryos from diabetic and non-diabetic mice, the estrous female diabetic/ non-diabetic mice were naturally mated with normal males, respectively. Vaginal plug was examined the next morning. The mice with a vaginal plug were killed and the pronuclear embryos were collected from their oviductal ampullae.

To transfer embryos to pseudopregnant mice which were produced by mating the normal estrous female mice with males with vasoligation, the pesudopregnant mice were anesthetized by intraperitoneal injection of sodium pentobarbital (40 mg/kg, Sigma). Then eight embryos were transferred into the oviduct of a pseudopregnant mouse. The mice were fed until gestational 10.5dpc and then killed. Embryos and placentas were collected.

### Statistical analysis

Data are presented as means ± SD. The methylation level of DMRs and expression levels of genes from different groups were compared by independent-sample t-test. A probability level of P < 0.05 was considered significant.

## Results

### Methylation patterns of imprinted genes’ DMRs in 10.5dpc placentas are affected by maternal diabetes mellitus

**Figure 1:**
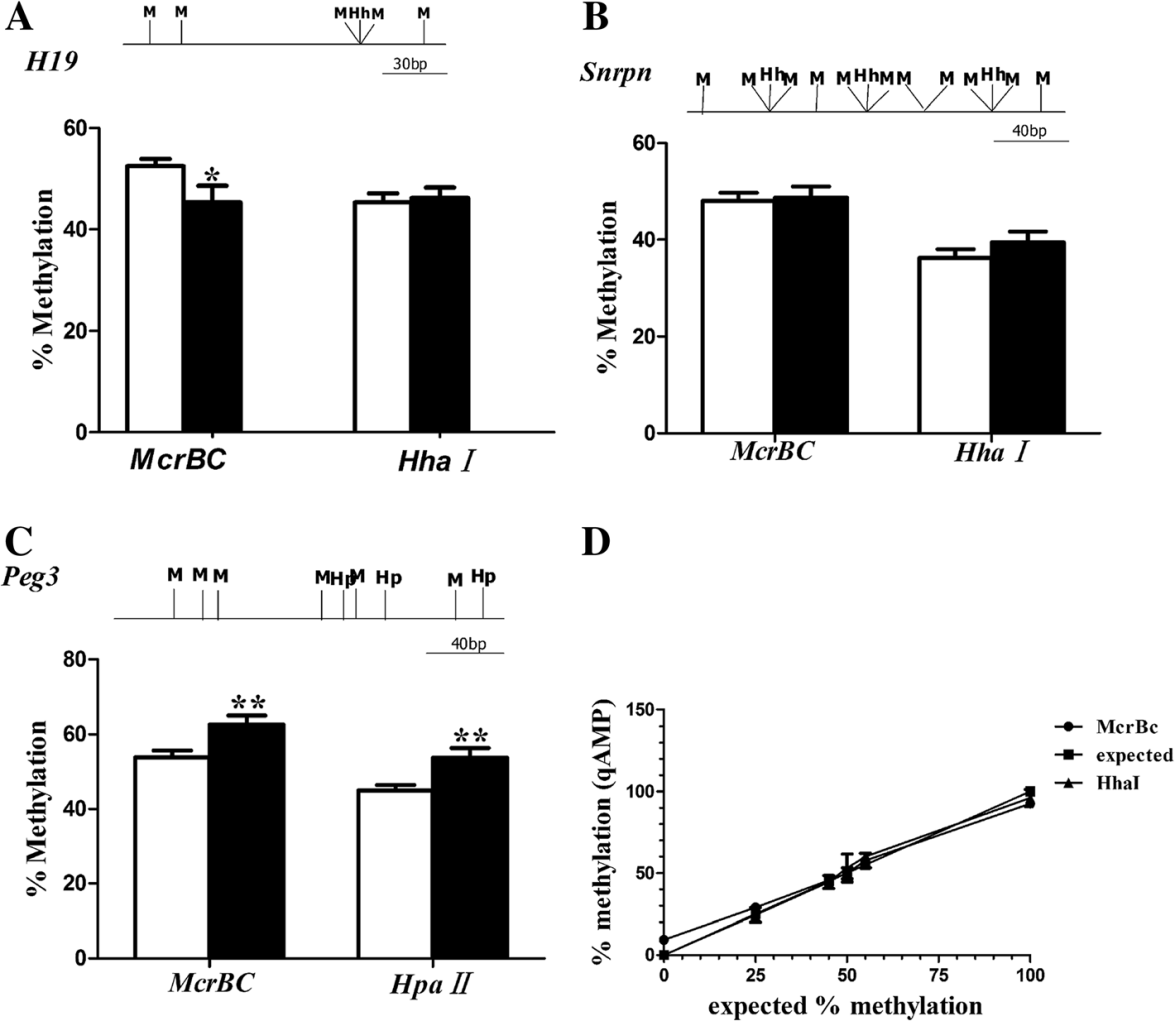
**Quantitative DNA methylation in DMRs of imprinted genes in 10.5dpc placentas as revealed by qAMP.** Diabetic (n = 26 from 5 litters) and nondiabetic (n = 25 from 5 litters) placentas at mid-gestation were recovered. **(A)** The paternally methylated *H19*; **(B, C)** Percentage methylation values at single and groups of restriction sites in the maternally methylated DMRs of *Snrpn* and *Peg3.* The lines represent the DMRs which were analyzed and the letter indicates the recognition sites of the enzymes. M, McrBc; Hh, HhaI; Hp, HpaII. **(D)** Confirmation of gene methylation level was determined by qAMP to expected values. HhaI and McrBc sites in chr9:106724005–106724149 are unmethylated in the genome. A primer pair flanking these sites was utilized to amplify DNA (NIH 3 T3 mouse genomic DNA and CpG methylated NIH 3 T3 mouse genomic DNA, NEB) mixed at the ratios: 100:0, 75:25, 55:45, 50:50, 45:55, 0:100. The qAMP values were close to expected values. Data are presented as mean ± SE for each enzyme employed. White bar, non-diabetic group; black bar, diabetic group; *P < 0.05, **P < 0.01.

To understand how maternal diabetes mellitus affects methylation levels of imprinted genes in 10.5dpc placentas from female diabetic mice mated with normal males within 15 days of STZ injection, qAMP was used to evaluate the average methylation levels of DMRs. For paternally imprinted gene *H19*, the average methylation levels were 43.92 ± 3.24% and 52.5 ± 1.42% in *McrBc* sites (*P* = 0.021), and 46.21 ± 2.06% and 45.34 ± 1.8% in *HhaI* sites in diabetic and nondiabetic groups (Figure 1A), respectively. At *HhaI/HpaII* sites, the mean methylation levels of *Snrpn* were 52.62 ± 2.44%, 39.44 ± 2.45% in the diabetic group and 47.32 ± 1.52%, 36.21 ± 1.81% in controls (Figure 1B), respectively. In placentas, the methylation levels of DMRs of maternally imprinted gene *Peg3* were significantly altered in diabetic mothers compared to controls. As shown in Figure 1C, the average methylation levels of *Peg3* in *McrBc* and *HpaII* sites were 62.66 ± 2.33%, 53.74 ± 2.61% and 53.82 ± 1.91%, 44.94 ± 1.49% in diabetic and non-diabetic groups, respectively.

To confirm that qAMP is sensitive enough to evaluate DNA methylation, we have chosen a DNA fragment in which all CpG sites were unmethylated. We determined that qAMP was sensitive enough to detect the DNA methylation status for imprinted gene DMRs (Figure 1D).

### The average methylation levels of imprinted genes are not altered in the fetus of diabetic females

**Figure 2:**
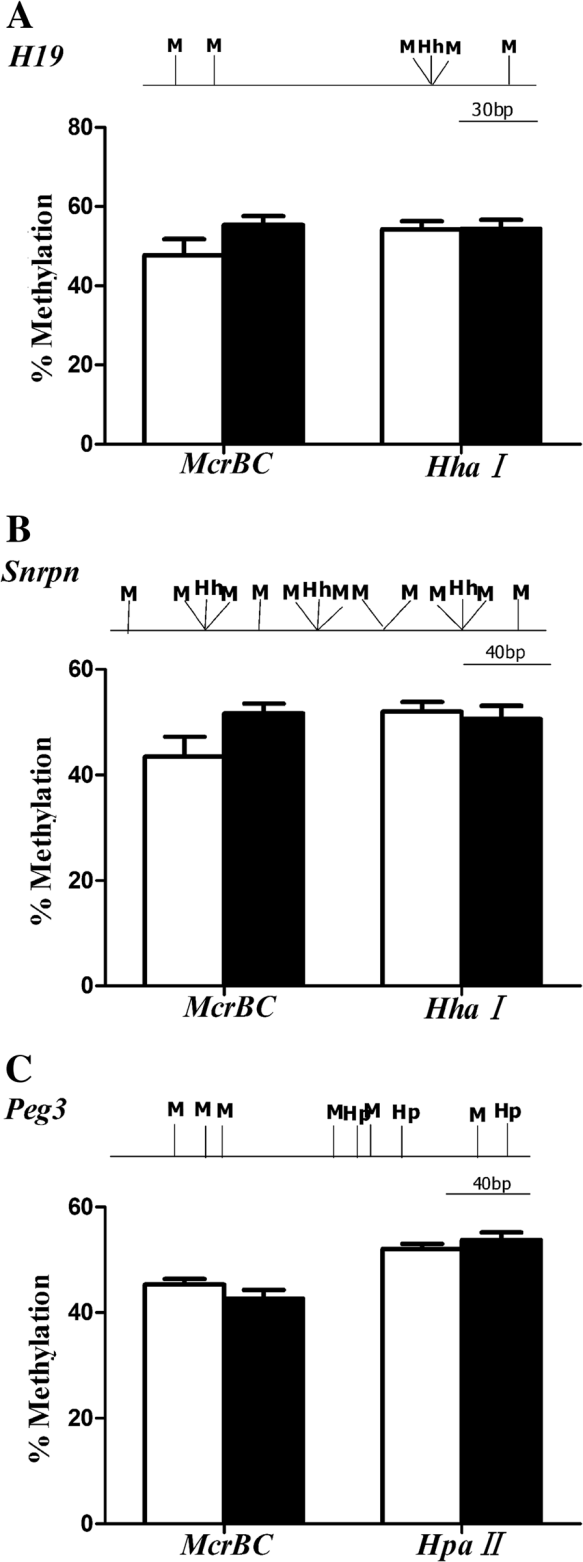
**Average DNA methylation levels in DMRs of imprinted genes in 10.5dpc fetus as revealed by qAMP.** Diabetic (n = 12 from 4 litters) and control (n = 12 from 4 litters) fetus were collected at 10.5dpc of gestation. DNA was digested with *Hha1*(Hh), *HpaII*(Hp) or *McrBC* (M) and amplified using real-time PCR. The locations of flanked restriction sites were displayed for each DMR. **(A)** Shown are the average methylation levels of paternally methylated gene H19 DMR; **(B, C)** represented the DNA methylation status in DMRs of *Snrpn* and *Peg3*. Data were presented as mean ± SE for each enzyme employed. White bar, non-diabetic group; black bar, diabetic group.

To investigate the methylation status of imprinted genes in dpc10.5 fetus, the methylation levels in DMRs of maternally imprinted genes *Peg3, Snrpn,* and paternally imprinted gene *H19* were determined by qAMP. The methylation pattern in DMR of *H19* was not affected by maternal diabetes in the fetus of female diabetic mice (Figure 2A). There was also no significant difference observed in the methylation patterns of *Snrpn* DMR between the diabetic and non-diabetic embryos (Figure 2B). The average methylation level of *Peg3* was similar between diabetic and non-diabetic groups when the samples were digested by *HpaII* and *McrBc* (Figure 2C).

### Expressions of *Peg3* and *H19* are altered by maternal diabetes in placentas but not in the fetus at mid-gestation

**Figure 3:**
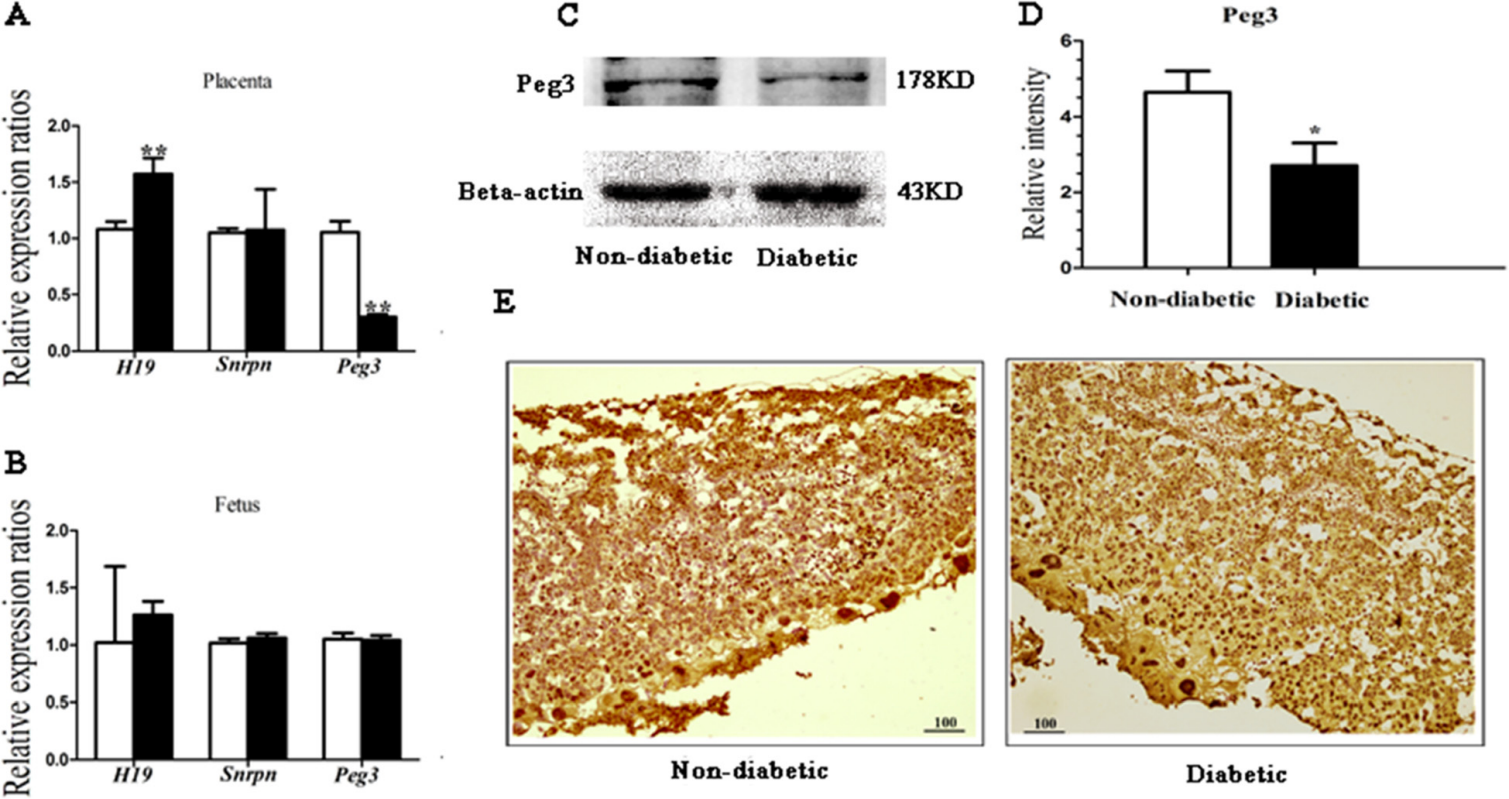
**Analysis of the mRNA and protein expression levels of imprinted genes in 10.5dpc placentas and fetus. (A, B)** Placentas (n = 30 from 6 litters for each group) and fetus (n = 12 from 4 litters for each group) were collected at 10.5dpc of gestation. Total RNA was purified and reverse transferred into cDNA and then amplified using qRT-PCR. **(A)** Relative expression levels of *H19, Snrpn* and *Peg3* in placentas. **(B)** Relative expression levels of *H19, Snrpn* and *Peg3* in fetus. **(C)** The protein expression of *Peg3* in placentas (n = 6) was investigated by western blot analysis and **(D)** the relative intensity of Peg3/beta-actin was evaluated by gel level analysis. **(E)** Placentas (n = 6) from diabetic and non-diabetic groups were stained by anti-Peg3 at 1:500 for histological analysis. Scale bar, 100 μm. Data are presented as mean ± SD. White bar, non-diabetic group; black bar, diabetic group; *P < 0.05, **P < 0.01.

We also assessed whether the expression levels of imprinted genes were affected by maternal diabetes in placentas and fetus at 10.5dpc by quantitative real-time PCR (qRT-PCR). For placentas, the expression levels of *Snrpn* were similar between the two groups, indicating that the expression was not disturbed by maternal diabetes mellitus. But expression levels of paternally imprinted gene *H19* and maternally imprinted gene *Peg3* showed significant alterations in the diabetic group compared to controls (Figure 3A). In the fetus, the expression levels of *Snrpn, Peg3* and *H19* in the diabetic groups were similar to controls (Figure 3B).

We also investigated the protein expression of *Peg3* in placentas. The result showed that the protein level was significantly lower (P = 0.039) in the diabetic group than in the control group (Figure 3C, D). And we found that *Peg3* was expressed in the placenta (Figure 3E) and the expression level in the control group was clearly higher than that in the diabetic group.

### Alterations of *Peg3* methylation and expression in the dpc10.5 placenta are observed in different litters

**Figure 4:**
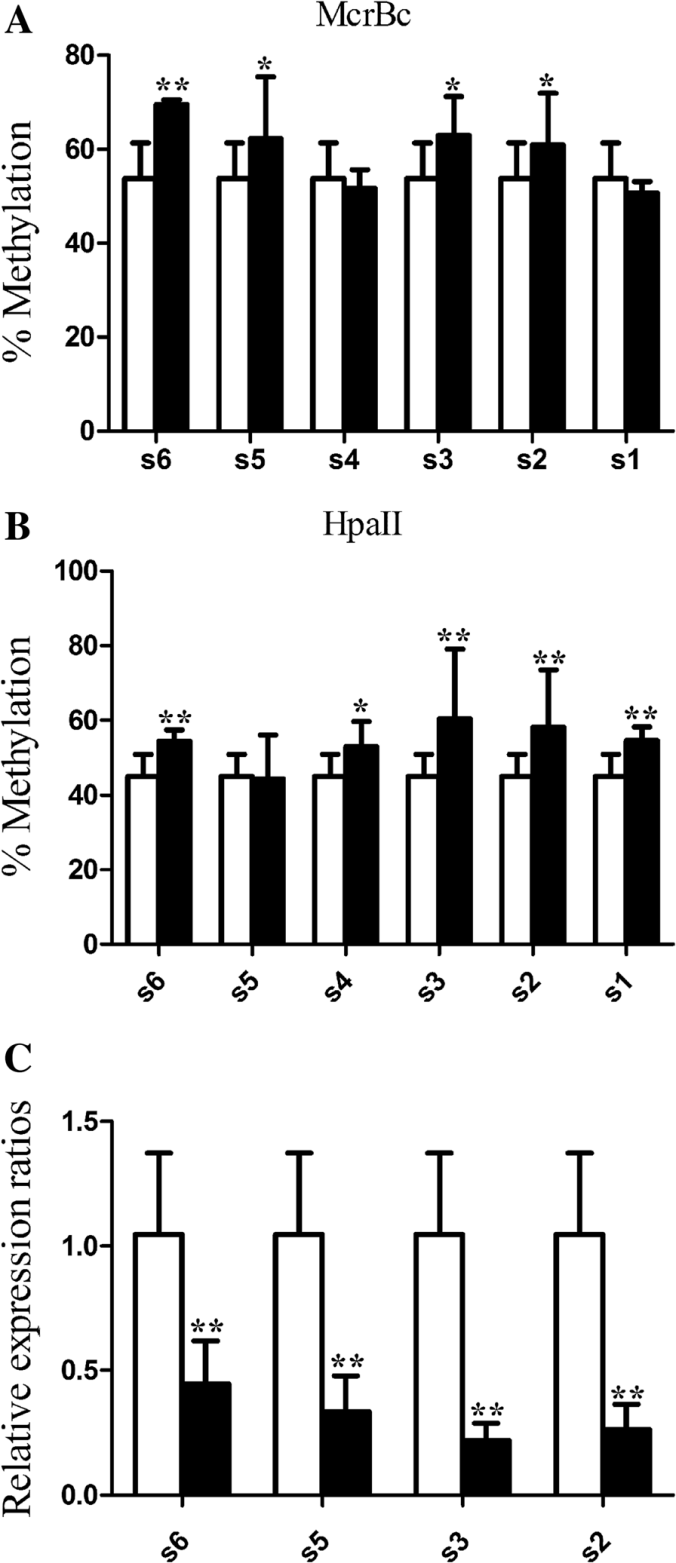
**The methylation level and expression of *Peg3 *in different litters. (A, B)** the methylation level of *Peg3* in different litters (n = 6 litters) at *McrBc* and *HpaII* was analyzed by qAMP; **(C)** the expression of *Peg3* in s2, s3, s5 and s6 was evaluated by qRT-PCR. White bar, nondiabetic; black bar, diabetic; number and letter under X-axis, litter; *P < 0.05; **P < 0.01.

We also investigated the methylation level and mRNA expression of *Peg3* in dpc10.5 placentas between different diabetic mothers. Six litters marked as s1-s6, respectively, were analyzed. For s2, s3 and s6, methylation levels at *McrBc* and *HpaII* were significantly higher compared to the nondiabetic mother (Figure 4A, B). The methylation levels of s1 and s4 were clearly higher at *HpaII* than the control (Figure 4B). At *McrBc*, the methylation level of s5 was increased in dpc10.5 placentas of diabetic females (Figure 4A). Meanwhile, we found that the expression of *Peg3* was significantly reduced by maternal diabetes mellitus for s2, s3, s5 and s6 (Figure 4C).

### The alterations of *Peg3* methylation and expression may mainly be caused by the adverse uterus environment

**Figure 5:**
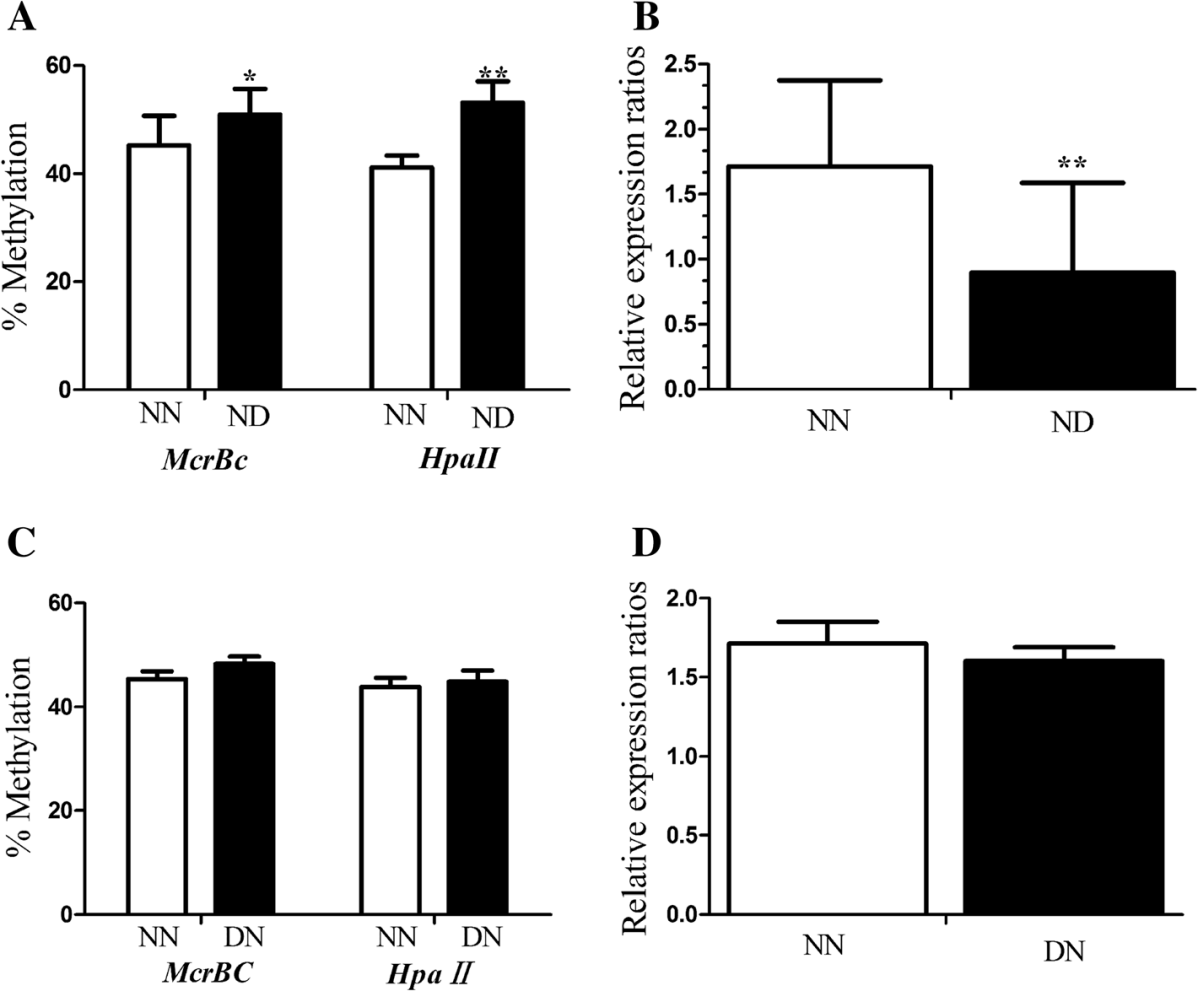
**DNA methylation and expression levels of *Peg3 *in placentas at mid-gestation. (A, B)** Non-diabetic pronuclear embryos were transferred to normal/diabetic (NN/ND) pseudopregnant female and the DNA methylation and expression levels of *Peg3* (n, NN:ND = 12:15) in 10.5d placentas were analyzed by qAMP and qRT-PCR, respectively. **(A)** methylation level of *Peg3* in dpc10.5 placenta; **(B)** expression of *Peg3* in placenta. **(C, D)** DN’s (n = 15 from 4 litters) and NN’s (n = 15 from 4 litters) placentas were collected at mid-gestation. DNA was digested with *HpaII*or *McrBC* and amplified using real-time PCR. **(C)** the methylation in DMRs of Peg3 in placentas; **(D)** Relative expression levels of *Peg3* in placentas. Data were presented as mean ± SD. *P < 0.05, **P < 0.01.

When pronuclear embryos of normal mothers were transferred to diabetic/normal pseudopregnant females (ND/NN), we found that the methylation level of *Peg3* was increased in ND (Figure 5A) placentas. Meanwhile, we observed that the mRNA expression of *Peg3* was reduced in ND (P < 0.01, Figure 5B) placentas.

When we transferred pronuclear embryos from diabetic/non-diabetic mice to normal pseudopregnant mice (DN/NN), the average methylation levels in DMRs of *Peg3* were similar between DN and NN in placentas (Figure 5C). The expression of *Peg3* in placentas of NN and DN was also similar (Figure 5D).

## Discussion

Proper establishment and maintenance of DNA methylation patterns are crucial for embryo development and survival. In the present study, we investigated whether maternal diabetes mellitus could perturb the acquisition/maintenance of DNA methylation during embryo development. In our study, we found that the expressions of *Peg3* and *H19* were altered in placentas at 10.5dpc; we also identified evident changes in the methylation level in the DMRs of them. Strikingly, these effects were not observed when diabetic pronuclear embryos were transferred into non-diabetic recipients.

The placenta is not only the specialized organ used by the developing embryo and fetus to obtain nutrients and oxygen from the mother, but it also acts as a key selectively permeable barrier between the mother and the fetus [29]. In pregnancies complicated by pre-gestational diabetes (type 1 and type 2), the *de novo* synthesis of blood vessels in the placenta shows abnormalities [30]. Several studies show that the expressions of leptin, leptin receptors, androgen receptor and peroxisome proliferator-activated receptors (PPARs) are altered by maternal diabetes mellitus in placentas [31,32]. In our study, we also observed that the mRNA levels of *H19* and *Peg3* were significantly altered in placentas from diabetic females compared to controls. These imprinted genes are expressed monoallelically in a parent-of-origin specific manner. They are generally located in clusters, epigenetically marked by DNA methylation on differentially methylated regions (DMRs) which regulate relative genes’ expression [33-35]. Interestingly, there were significant changes of average methylation levels in DMRs of *H19* and *Peg3* in placentas from diabetic mice. It is well known that DNA methylation is a mechanism regulating expression of imprinted genes. Our results indicate that abnormal methylation levels may be a reason for the changed expressions of these genes. However, these changes might be induced by different distribution of *Peg3* in different placental cell populations. Hiby et al. found that *Peg3* was expressed in the trophoblast of the developing placenta at 9 days post-coitum (d.p.c.) and the trophoblast populations of the well-developed placenta at 15 dpc [36]. In our study, the placenta was separated and used to carry out all the relative assays. By immunohistochemistry analysis, we observed that all the trophoblast populations were stained. The Western blot analysis also indicated that the protein level of *Peg3* in diabetic placentas was obviously lower. These findings might partly elucidate how maternal diabetes mellitus induces abnormal embryo development, because proper DNA methylation patterns are important for placenta and fetus development.

Previous studies found that the diabetic condition is detrimental to pre- and post-implantation embryo development. Congenital malformations are approximately 3–4 times more frequent in infants from diabetic mothers than from non-diabetic mothers [37,38]. In both chemically induced and spontaneous diabetic models, significant abnormalities in pre-implantation embryo development have been observed [6,39,40]. But the DNA methylation patterns in fetus from diabetic females are still unclear. In our study, we examined the expression and average methylation levels of some imprinted genes in the placenta and embryo at 10.5dpc. We found that the expression of relative imprinted genes was not altered in diabetic dpc10.5 fetus compared with controls. The expression of *H19* was consistent with that reported by Shao *et al.*[41]. The average methylation levels in DMRs of them were also similar to controls. Although another study found that the methylation level of *H19-Igf2* imprint control region was increased in the embryonic day 14 fetus from diabetic mice [41], we found that the methylation level of *H19* DMR was not affected by maternal diabetes in the 10.5dpc embryo. Different methods employed in these studies may be a reason for the difference in results. Another reason may relate to the fact that we selected different fragments as targets. Because severe loss of DNA methylation in imprinted genes causes embryo absorption and miscarriage during early development, it was not possible to identify significant loss of DNA methylation in DMRs of imprinted genes in live fetus from diabetic females. Another reason may be the protective function of the placenta which serves as filter between mother and fetus. In our study, we found no changes of imprinted genes in fetus from mothers with diabetes mellitus, but the expression and methylation levels were significantly affected by maternal diabetes in placentas. These findings suggest that maternal diabetes may affect fetus development by altering placental functions.

Studies have shown that if maternal diabetes mellitus is well controlled, the deleterious effects on embryo development can be partially corrected. Studies also have shown that when the diabetic females are treated with insulin, embryo development is not significantly different from non-diabetic females [6]. These results indicate that the diabetic uterus environment may play a key role in abnormal embryonic development. So we transferred pronuclear embryos of normal females to normal/diabetic pseudopregnant females and found that the DNA methylation patterns and expression of *Peg3* in dpc10.5 placentas were significantly changed by the diabetic uterus environment. However, while pronuclear embryos of diabetic mothers were transferred to normal pseudopregnant females (DN), the expression and methylation levels of *H19* and *Peg3* in placentas were similar between DN and NN. Another study has demonstrated that these genes’ methylation status in oocytes of diabetic females was not affected [23] at 15 days of injection of STZ. So we propose that the altered expression and methylation levels of imprinted genes may primarily be caused by the adverse uterus environment of diabetic females and it could be corrected by embryo transfer.

## Conclusions

In summary, our data show that the expression of imprinted genes is disturbed by maternal diabetes mellitus in mid-gestation and this may be caused by altered methylation patterns in DMRs. Such effect could be corrected by transfer of pronuclear embryos from a diabetic mother into a normal uterus. In addition, when pronuclear embryos of normal females were transferred to diabetic pseudopregnant females, the alterations of imprinted genes were also observed. Therefore, the detrimental maternal diabetic uterus environment may play a key role in causing the deleterious effects on imprinted genes in the dpc10.5 placenta. Our evaluations of methylation and expression of imprinted genes were only carried out in live mid-gestation embryos and several DMRs. The conditions regarding the entire genomic imprinting pattern still need further clarification.

## Competing interests

The authors declare that they have no competing interests.

## Authors’ contributions

ZJG carried out the epigenetic analysis, designed the assay and wrote the manuscript. QXL carried out the epigenetic analysis. SML, YCW and ZJG carried out PCR amplification and epigenetic analysis. HZM participated in analyzing the data. HS participated in revising the manuscript. QYS and CLZ participated in the design of the assay and revising the manuscript. All authors read and approved the final manuscript.
